# Predictive Value of TyG and TyG-BMI Indices for Non-Alcoholic Fatty Liver Disease in High-Altitude Regions of China: A Cross-Sectional Study

**DOI:** 10.3390/jcm13237423

**Published:** 2024-12-05

**Authors:** Xuejie Wang, Ziqiu Wang, Wen Du, Xiaobo Ma, Jun Ma, Zijin Chen, Chenni Gao, Xiaonong Chen

**Affiliations:** 1 Department of Nephrology, Ruijin Hospital Lu Wan Branch, Shanghai Jiao Tong University School of Medicine, Shanghai 200025, China; 2 Department of Nephrology, Ruijin Hospital, Shanghai Jiao Tong University School of Medicine, Shanghai 200025, China; mxb11598@rjh.com.cn (X.M.);

**Keywords:** triglyceride-glucose index, body mass index, non-alcoholic fatty liver disease, high-altitude regions, receiver operating characteristic curve

## Abstract

**Background:** The associations between triglyceride glucose (TyG), triglyceride glucose-body mass index (TyG-BMI), and non-alcoholic fatty liver disease (NAFLD) in high-altitude regions remain unclear. **Methods:** This is a cross-sectional, population-based study comprising 1384 adults living in Jianchuan county, China, which has an average altitude of over 2200 m. Logistic regressions were used to examine the associations between TyG, TyG-BMI, and NAFLD. Receiver operating characteristic (ROC) curves were utilized to compare the predictive ability of TyG, TyG-BMI, hepatic steatosis index (HSI), and triglyceride glucose-alanine aminotransferase (TyG-ALT). **Results:** In total, 307 (35.7%) male and 81 (15.4%) female participants were diagnosed with NAFLD. Individuals with NAFLD had higher BMI, blood pressure, and TyG indices. The adjusted odds ratios (95% confidence intervals) for the highest quartile of TyG and TyG-BMI were 16.04 (8.51–30.25) and 48.55 (25.12–93.81), respectively. The areas under the ROC curve were 0.811 (95% CI: 0.787–0.836) for TyG, 0.883 (95% CI: 0.864–0.902) for TyG-BMI, 0.839 (95% CI: 0.817–0.863) for HSI, and 0.831 (95% CI: 0.801–0.855) for TyG-ALT. Tyg-BMI had the highest sensitivity (0.832) and specificity (0.780) compared to the other indices. **Conclusions:** Both TyG and TyG-BMI were associated with higher NAFLD risk in people living in high-altitude regions, while TyG-BMI had greater predictive capabilities.

## 1. Introduction

Non-alcoholic fatty liver disease (NAFLD) is the most common chronic liver disease worldwide, with a prevalence of 30.05% in 2020 [1]. NAFLD is closely linked to metabolic dysfunction and a higher risk of liver fibrosis [2,3]. Various approaches have been developed to explore effective tools for predicting and monitoring NAFLD [4]. Among several predictive indices for NAFLD, the triglyceride glucose (TyG) index has emerged as a promising biomarker due to its simplicity and accessibility. It is a robust surrogate marker for insulin resistance (IR), which is pivotal in the pathogenesis of NAFLD [5]. According to previous studies, TyG was associated with a 1.8- to 4.7-fold increase in the risk of NAFLD [6,7,8]. More recently, several TyG-related indices have been developed. The combination of TyG index with body mass index (BMI) and waist circumferences has been proposed to offer enhanced predictive accuracy for NAFLD, compared with simple TyG [9,10]. For example, it is suggested that TyG-BMI has a higher predictive ability than the hepatic steatosis index and many other indices [11,12].

Although the studies referred to above highlighted the potential utility of TyG and TyG-related parameters in assessing the risk of NAFLD, all of them were conducted in low-altitude areas. In high-altitude regions, physiological responses to hypoxia, dietary limitations, and unique lifestyle factors can influence both the TyG index and NAFLD development [13]. Specifically, hypoxia may promote insulin resistance, lipid accumulation, and oxidative stress, contributing to a higher TyG index and NAFLD susceptibility. Additionally, altitude-specific lifestyle factors, such as high physical activity levels and high-protein dietary preference, might either mitigate or exacerbate these risks [14,15]. Despite all these characteristics, the associations between TyG-related indices and NAFLD in residents living in high-altitudes have not been investigated previously. To address the knowledge gaps, we conducted a population-based study in Jianchuan county, Yunnan province, China [16]. Jianchuan has an average altitude of over 2200 m and is a suitable location for exploring whether TyG-related indices and NAFLD are associated in high-altitude areas. Moreover, we compared the predictive ability of TyG-related indices with two other well-recognized hepatic indices, namely, hepatic steatosis index (HSI) [17] and triglyceride glucose-alanine aminotransferase (TyG-ALT) [18].

## 2. Materials and Methods

### 2.1. Study Design

This cross-sectional, population-based study was conducted in the People’s Hospital of Jianchuan County between January and December 2019. In total, 1552 Bai individuals receiving medical examinations were asked to participate. Those who were younger than 18 years (*n* = 30), currently pregnant (*n* = 2), or previously diagnosed with malignant tumors (*n* = 48), with viral/autoimmune hepatitis (*n* = 79) or with excessive alcohol consumption (defined as alcohol intake ≥30 g/day for men and ≥20 g/day for women) (*n* = 9) were excluded [19]. A total of 1384 participants were included in the final analysis. A history of previous liver disease was obtained from the patient’s medical records.

### 2.2. Data Collection

Data were collected according to the standard procedures of the hospital. Demographic data, including age, sex, height, and weight, were collected on site. Participants’ heights and weights were carefully measured twice, without shoes and heavy clothes, using the HNH-219 automatic height/weight measurement unit (Omron, Kyoto, Japan). The average of the two readings was recorded as the corresponding height and weight. The BMI was calculated as the body weight divided by the square of the height. Venous blood samples were drawn by trained medical professionals after an overnight fast for 8 h before sample collection. No specific instructions were given regarding diet or physical activity prior to the examination. All samples were subsequently analyzed at the local hospital’s laboratory. Complete blood count was tested using an Auto Hematology Analyzer BC-5000. Creatinine, uric acid, fasting plasma glucose (FPG), serum albumin, total cholesterol (TC), triglyceride (TG), high-density lipoprotein cholesterol (HDL-c), low-density lipoprotein cholesterol (LDL-c), alanine aminotransaminase (ALT), and aspartate aminotransferase (AST) were tested at local accredited laboratories. These biochemical indices were measured on a LABOSPECT 008 AS automated analyzer (Hitachi, Ibaraki-ken, Japan). Each participant received an abdominal ultrasound examination scan. All participants provided signed informed consent, and their details were all explained. As this was a retrospective cross-sectional study, we received an exemption of ethics board approval from the Ethics Committee of Ruijin Hospital, Shanghai Jiao Tong University, School of Medicine (Shanghai, China).

### 2.3. Diagnosis of NAFLD

Ultrasound is the first-line imaging method in clinical practice for assessing suspected NAFLD [20]. A color Doppler ultrasound machine (model IU22, Philips Healthcare, Andover, MA, USA) equipped with a 1.0–5.0 MHz transducer was used for conducting abdominal ultrasound scans in the current study. To ensure the inter-rater reliability of the diagnosis, two experienced radiologists independently performed the ultrasound examinations and assessed the liver echogenicity in a blinded manner. For any discrepant cases, a third senior radiologist was available to review and reach a final consensus diagnosis. However, no discrepancy cases were identified in the current study. Therefore, we did not refer to the third expert. Diagnosis was made based on higher echogenicity of liver tissue and reduced clarity of vascular structures. Quantitative criteria included the following two conditions [21]:

Liver-to-kidney echogenicity ratio >1.15 indicates hyperechoic liver with increased echogenicity.

Intrahepatic vessel clarity scores falling below “1” indicate poor visibility of intrahepatic vascular structures.

### 2.4. Definitions of Exposures and Other Covariates

TyG, TyG-BMI, HSI, and TyG-ALT were calculated according to the following Algorithm 1.
**Algorithm 1** Formula to Calculate Hepatic Indices: TyG, TyG-BMI, TyG-ALT and HSITyG = Ln [TG (mg/dL) × FPG (mg/dL)/2]; TyG-BMI = TyG × BMI (kg/m^2^); TyG-ALT = Ln (TG × FPG)×ALT (IU/L); HSI = 8 × AST/ALT + BMI + 2 (if female) + 2 (if diabetes), with diabetes defined as an FPG > 7.0 mmol/L [22].

Abbreviations used in Algorithm 1: TyG, triglyceride-glucose index; TG, triglycerides; FPG, fasting plasma glucose; BMI, body mass index; HSI, hepatic steatosis index; ALT, alanine aminotransferase; AST, aspartate aminotransferase.

Covariates included in the adjustment model are age, gender, BMI (not for TyG-BMI), systolic blood pressure [23], and hemoglobin. Hemoglobin is included since it partly reflects the unique physiological characteristic of individuals living in high-altitudes [24].

### 2.5. Statistical Analysis

Continuous variables are described as the mean ± standard deviation (SD), and the Shapiro–Wilk test was used to assess the normality of the data. For normally distributed variables, an independent sample *t*-test was used to compare differences between the NAFLD and non-NAFLD groups; for variables that were not normally distributed, the Mann–Whitney U-test was applied. A *p*-value of less than 0.05 was considered statistically significant.

To evaluate the association between the TyG index, TyG-BMI, and the risk of NAFLD, logistic regression analysis was employed. TyG index and TyG-BMI were divided into four quartiles, with the first quartile (Q1) serving as the reference group. Receiver operating characteristic (ROC) curves were used to assess the predictive ability of the TyG, TyG-BMI, HSI, and TyG-ALT for NAFLD, and the areas under the ROC curve (AUC) were calculated. The optimal cutoff point, where sensitivity and specificity were highest, was determined using the Youden index method. Differences between the curves were compared using the DeLong test.

All data analyses were conducted using R software (version 4.4.1). A *p*-value less than 0.05 was considered statistically significant.

## 3. Results

### 3.1. Demographic Characteristics and Laboratory Results of Participants

In total, 1384 participants were enrolled in this study, comprising 859 (62.1%) males and 525 (37.9%) females, with an average age of 42.4 years. Of them, 388 had NAFLD, consisting of 307 males and 81 females (*p* < 0.001). Compared to the non-NAFLD group, participants in the NAFLD group were significantly older and had a higher level of BMI, blood pressure, ALT, AST, serum creatinine, uric acid, fasting glucose, total cholesterol, triglyceride, HDL-c, and LDL-c. The mean (SD) of the TyG index and TyG-BMI was 9.14 ± 0.67 and 246.13 ± 30.32 (P for t-test < 0.001) within the NAFLD group and 8.43 ± 0.58 and 195.61 ± 30.42 in the non-NAFLD group (P for t-test < 0.001), respectively (Table 1).

### 3.2. Associations Between TyG, TyG-BMI, and NAFLD

Both unadjusted and adjusted logistic regression models were employed to assess the association between the TyG index, TyG-BMI, and NAFLD risk. As shown in Table 2, the unadjusted odds ratios (ORs) increased progressively across the TyG quartiles, with participants in the highest quartile (Q4) having significantly higher odds of NAFLD compared to the lowest quartile (Q1) (odds ratio (OR): 40.39; 95% CI: 22.67–71.96). Similar trends were observed for TyG-BMI, where the odds of NAFLD in the highest quartile (Q4) were strikingly elevated (OR: 63.63; 95% CI: 33.50–120.84).

After adjusting for potential confounders, the association remained significant. For the TyG index, the highest quartile (Q4) was associated with a 16.04-fold increased risk of NAFLD (adjusted OR: 16.04; 95% CI: 8.51–30.25), while TyG-BMI in Q4 retained a high adjusted OR of 48.55 (95% CI: 25.12–93.81).

### 3.3. ROC Curve Analysis of Hepatic Indices in Assessing NAFLD

**Figure 1:**
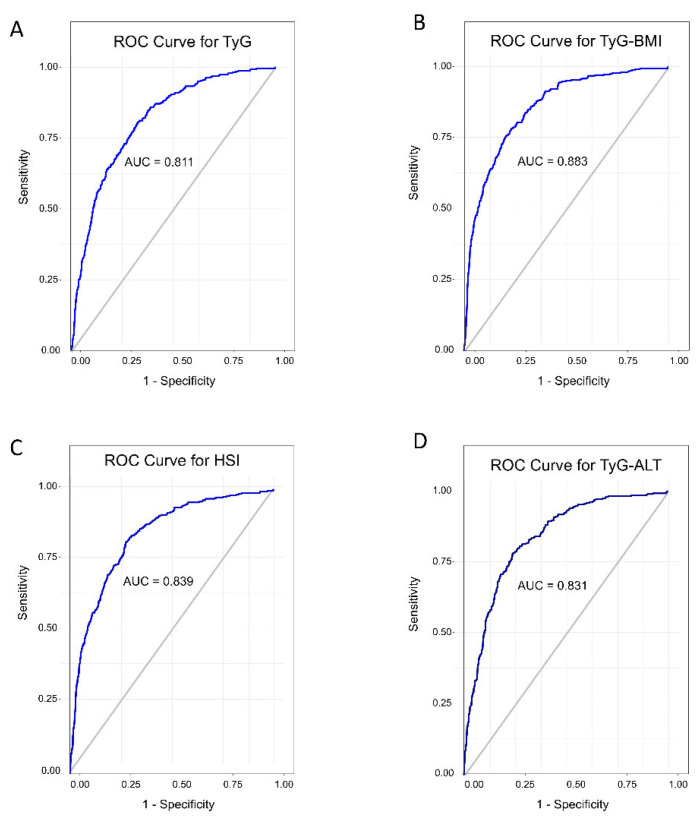
ROC curves comparing diagnostic performance of TyG, TyG-BMI, HSI, and TyG-ALT for predicting NAFLD. ROC curves comparing diagnostic performance of TyG, TyG-BMI, HSI, and TyG-ALT for predicting NAFLD, (**A**): ROC curves for TyG; (**B**): adjusted ROC curves for TyG-BMI; (**C**): ROC curves for HIS; (**D**): ROC curves for TyG-ALT.

As shown in Table 3, TyG, TyG-BMI, HSI, and TyG-ALT all demonstrated a compelling ability to predict NAFLD. The AUC for the TyG index was 0.811 (95% CI: 0.787–0.836), with an optimal cutoff value of 8.63 for assessing NAFLD, yielding a sensitivity of 80.6%, a specificity of 67.5%, and a Youden index of 0.481. Compared to TyG, TyG-BMI exhibited higher predictive accuracy for NAFLD, with an AUC of 0.883 (95% CI: 0.864–0.902); the sensitivity and specificity were 83.2% and 78.0%, respectively. The Youden index was 0.612 at the optimal cutoff of 218.08. The AUC was 0.839 for HSI and 0.831 for TyG-ALT. These results suggested that all the hepatic indices are effective in predicting NAFLD, with TyG-BMI showing the best diagnostic performance (Delong test *p* < 0.001). ROC curves for all the indices in predicting NAFLD are depicted in Figure 1.

## 4. Discussion

This study demonstrates that both the TyG and TyG-BMI indices are strongly linked to an increased risk of NAFLD. In the adjusted analysis, participants in the highest quartile for the TyG index were found to have a 16.04-fold risk of NAFLD, compared to those in the lowest quartile. Similarly, the risk for individuals in the highest TyG-BMI quartile was 48.55 times higher. The analysis shows that the TyG index performed well in predicting NAFLD with an AUC of 0.811, and TyG-BMI demonstrated even greater accuracy with an AUC of 0.883.

TyG and related indices are strongly associated with an elevated risk of metabolic dysfunction and cardiovascular disease [25,26,27]. More recently, it has been further related to a higher risk of NAFLD and liver fibrosis in China and many other countries [28,29,30]. Although TyG demonstrates predictive capability for NAFLD across ethnicities, previous studies have mainly been conducted among individuals living in plains or low-altitude areas. It is worth noting that residents in high-altitude areas show significantly different metabolic patterns to those living in plains, especially in terms of lipid metabolism and body composition [31,32]. Therefore, it is unclear in the literature whether TyG can still serve as a reliable indicator to predict NAFLD risk. In the current study, 1384 participants living in Jianchuan county, an area with an average altitude of over 2200 m, were enrolled. Within this special subgroup, we confirmed the association between TyG and NAFLD in those living in high-altitudes. We also noticed that the odds ratio of NAFLD for TyG and TyG-BMI are significantly higher than those in low-altitude areas [33,34]. This observation further indicates that altitude variation impacts the association between TyG indices and NAFLD. Of all the hepatic indices, TyG-BMI proved to have the highest AUC and Youden index, compared to TyG and traditional NAFLD indices (TyG-ALT and HSl). This is in line with several previous studies [35,36]. It is speculated that, since obesity is the driving force of NAFLD [37], combining these two variables might help to improve the specificity and sensitivity of the diagnosis of NAFLD. Despite the recent transition from NAFLD to MASLD terminology [38], the current dataset lacked information on several metabolic variables and medication use. Therefore, we were unable to conduct a comprehensive evaluation using the current MASLD criteria. Future studies with complete metabolic profiling would provide more comprehensive insights into the relationship between TyG-related indices and steatotic liver diseases across different definitions.

The TyG index is intrinsically linked to the development of NAFLD, as well as its progression to NASH through multiple interconnected pathways [39]. An elevated TyG index reflects increased IR, which is fundamental in the pathogenesis of NAFLD and NASH [40]. Specifically, IR promotes lipolysis and results in an increased flux of free fatty acids to the liver [41]. Meanwhile, the accumulated lipids might exacerbate lipotoxicity and trigger systematic inflammation [42]. These IR-induced pathophysiological processes ultimately contribute to hepatocellular damage [43].

This study is the first to validate the efficacy of the TyG index and TyG-BMI in evaluating NAFLD in high-altitude populations, expanding their potential as reliable indicators of NAFLD in individuals from different backgrounds. However, this study has some limitations. Firstly, this is a cross-sectional study, which does not allow for the determination of causal relationships. Future longitudinal studies should be designed to better understand the dynamic relationship between these indices and NAFLD. Secondly, while ultrasonography offers advantages in terms of cost-effectiveness and non-invasiveness, it has inherent operator dependency and limited capability in the precise quantification of hepatic fat content. It is acknowledged that magnetic resonance imaging (MRI) demonstrates superior accuracy in hepatic fat quantification. Nevertheless, ultrasonography maintains its position as a primary screening modality in clinical settings, given its established role in primary healthcare facilities [44,45]. Thirdly, there may be some unmeasured confounders, such as unique dietary habits and lifestyle characteristics in Bai individuals. Although we did not have specific data on these factors, we believe that our adjustments using hemoglobin provide an indirect means to account for some of the high-altitude-specific physiological factors that may influence NAFLD and the TyG index. We recognize that future studies could benefit from the collection of more detailed lifestyle and dietary information to further refine these findings. Lastly, studies based on health check-up populations may introduce selection bias.

## 5. Conclusions

This study is the first to validate the efficacy of TyG and TyG-BMI in predicting NAFLD in high-altitude populations, with TyG-BMI showing superior predictive performance. Considering the lack of specific diagnostic equipment in these areas, TyG and TyG-BMI might offer a cost-effective alternative of NAFLD detection. The application of TyG and TyG-BMI indices into routine clinical assessments will make early NAFLD screening more accessible in high-altitude regions.

## Figures and Tables

**Table 1:** Baseline characteristics of participants according to NAFLD status.

	Overall (*n* = 1384)	Non-NAFLD (*n* = 996)	NAFLD (*n* = 388)	*p* Value
Female N (%)	525 (37.9%)	444 (44.6%)	81 (20.9%)	<0.001
Age (yrs)	42.4 ± 11.7	41.4 ± 12.0	44.8 ± 10.5	<0.001
BMI (kg/m^2^)	24.2 ± 3.3	23.1 ± 2.9	27.0 ± 2.8	<0.001
SBP (mmHg)	121 ± 15	119 ± 15	126 ± 14	<0.001
DBP (mmHg)	76 ± 11	74 ± 11	81 ± 11	<0.001
Urea (mmol/L)	5.47 ± 2.81	5.41 ± 2.83	5.61 ± 2.78	0.285
Creatinine (μmol/L)	70.1 ± 20.8	68.6 ± 22.2	74.0 ± 15.9	<0.001
Uric acid (mmol/L)	347.9 ± 90.4	327.0 ± 83.9	401.5 ± 84.4	<0.001
Total cholesterol (mmol/L)	5.30 ± 1.07	5.16 ± 1.00	5.66 ± 1.16	<0.001
Triglyceride (mmol/L)	1.73 ± 1.59	1.41 ± 1.29	2.57 ± 1.95	<0.001
LDL-c (mmol/L)	3.14 ± 1.87	2.97 ± 1.46	3.58 ± 2.60	<0.001
HDL-c (mmol/L)	1.67 ± 0.35	1.69 ± 0.35	1.60 ± 0.34	<0.001
Fasting glucose (mmol/L)	5.16 ± 1.24	4.96 ± 0.81	5.69 ± 1.85	<0.001
Albumin (g/L)	49.6 ± 15.9	49.8 ± 18.6	49.1 ± 3.8	0.422
ALT (IU/L)	28.1 ± 20.8	23.9 ± 15.0	38.7 ± 28.4	<0.001
AST (IU/L)	26.9 ± 15.8	25.6 ± 10.8	30.3 ± 23.9	<0.001
TyG index	8.63 ± 0.68	8.43 ± 0.58	9.14 ± 0.67	<0.001
TyG-BMI	209.77 ± 37.92	195.61 ± 30.42	246.13 ± 30.32	<0.001

Abbreviations: BMI, body mass index; SBP, systolic blood pressure; DBP, diastolic blood pressure; LDL-c, low-density lipoprotein cholesterol; HDL-c, high-density lipoprotein cholesterol; ALT, alanine aminotransaminase; AST, aspartate aminotransferase.

**Table 2:** Associations between TyG, TyG-BMI, and NAFLD.

**TyG Index**	**Case (%)**	**Model 1**	**Model 2**
Q1	14 (4.05)	1.00 (ref)	1.00 (ref)
Q2	50 (14.45)	4.01 (2.17–7.40)	2.41 (1.24–4.68)
Q3	106 (30.64)	10.47 (5.86–18.74)	4.57 (2.42–8.61)
Q4	218 (63.01)	40.39 (22.67–71.96)	16.04 (8.51–30.25)
**TyG-BMI**	**Case (%)**	**Model 1**	**Model 2**
Q1	11 (3.18)	1.00 (ref)	1.00 (ref)
Q2	38 (10.98)	3.76 (1.89–7.48)	3.26 (1.63–6.54)
Q3	105 (30.35)	13.27 (6.98–25.23)	11.13 (5.79–21.40)
Q4	234 (67.63)	63.63 (33.50–120.84)	48.55 (25.12–93.81)

Model 1: crude model. Model 2: adjusted for age, gender, body mass index (only for TyG), systolic blood pressure, hemoglobin, and alanine aminotransaminase.

**Table 3:** Performance metrics of ROC curves for various hepatic indices.

	AUC	95% CI	SE	Optimal Cutoff	Sensitivity	Specificity	Youden Index
TyG	0.811	0.787–0.836	0.013	8.63	0.806	0.675	0.481
TyG-BMI	0.883	0.864–0.902	0.009	218.08	0.832	0.780	0.612
HSI	0.839	0.817–0.863	0.011	33.96	0.814	0.629	0.443
TyG-ALT	0.831	0.808–0.855	0.012	12.02	0.781	0.660	0.441

CI, confidence interval; SE, standard error; AUC, area under the ROC curve.

## Data Availability

The data presented in this study are available on request from the corresponding author due to reasonable request.
